# Maximizing Laboratory Production of Aflatoxins and Fumonisins for Use in Experimental Animal Feeds

**DOI:** 10.3390/microorganisms10122385

**Published:** 2022-11-30

**Authors:** Phillis E. Ochieng, David C. Kemboi, Marie-Louise Scippo, James K. Gathumbi, Erastus Kangethe, Barbara Doupovec, Siska Croubels, Johanna F. Lindahl, Gunther Antonissen, Sheila Okoth

**Affiliations:** 1 Laboratory of Food Analysis, Department of Food Sciences, Faculty of Veterinary Medicine, University of Liège, 4000 Liège, Belgium; 2 Department of Pathobiology, Pharmacology and Zoological Medicine, Faculty of Veterinary Medicine, Ghent University, 9820 Merelbeke, Belgium; 3 Department of Animal Science, Chuka University, Chuka P.O. Box 109-00625, Kenya; 4 Department of Veterinary Pathology, Microbiology and Parasitology, Faculty of Veterinary Medicine, University of Nairobi, Nairobi P.O. Box 29053-00100, Kenya; 5 Independent Researcher, Nairobi P.O. Box 34405-00100, Kenya; 6 DSM-BIOMIN Research Center, 3430 Tulln, Austria; 7 Department of Biosciences, International Livestock Research Institute (ILRI), Nairobi P.O. Box 30709-00100, Kenya; 8 Department of Medical Biochemistry and Microbiology, Uppsala University, 582 Uppsala, Sweden; 9 Department of Clinical Sciences, Swedish University of Agricultural Sciences, 7054 Uppsala, Sweden; 10 Chair Poultry Health Sciences, Faculty of Veterinary Medicine, Ghent University, 9820 Merelbeke, Belgium; 11 Department of Biology, Faculty of Science and Technology, University of Nairobi, Nairobi P.O. Box 30197-00100, Kenya

**Keywords:** *Aspergillus flavus*, *Fusarium verticillioides*, aflatoxins production, fumonisin production, mycotoxins, food safety, feed safety

## Abstract

Warm and humid climatic conditions coupled with poor agricultural practices in sub-Saharan Africa favor the contamination of food and feed by *Aspergillus flavus* and *Fusarium verticillioides* fungi, which subsequently may produce aflatoxins (AFs) and fumonisins (FBs), respectively. The growth of fungi and the production of mycotoxins are influenced by physical (temperature, pH, water activity, light and aeration), nutritional, and biological factors. This study aimed at optimizing the conditions for the laboratory production of large quantities of AFs and FBs for use in the animal experiments. *A. flavus* and *F. verticillioides* strains, previously isolated from maize in Kenya, were used. Levels of AFB1 and total FBs (FB1, FB2, and FB3) in different growth substrates were screened using ELISA methods. Maize kernels inoculated with three different strains of *A. flavus* simultaneously and incubated at 29 °C for 21 days had the highest AFB1 level of 12,550 ± 3397 μg/kg of substrate. The highest level of total FBs (386,533 ± 153,302 μg/kg of substrate) was detected in cracked maize inoculated with three different strains of *F. verticillioides* and incubated for 21 days at temperatures of 22–25 °C in a growth chamber fitted with yellow light. These two methods are recommended for the mass production of AFB1 and FBs for animal feeding trials.

## 1. Introduction

Mycotoxins are secondary metabolites synthesized by some species of toxigenic fungi that contaminate food and feed while in the field, during transportation, processing, or while in storage. In Sub-Saharan Africa (SSA), aflatoxins (AFs) and fumonisins (FBs) are reported to be the major mycotoxins occurring in the food and feed commodities [1,2]. Naturally, *A. flavus* produces AFB1 and AFB2, whereas *A. parasiticus* produces the four major aflatoxin types (AFB1, AFB2, AFG1, and AFG2) [3]. Aflatoxin-producing *A. flavus* strains have been recovered in feed and feed ingredients such as maize, sunflower, and peanut from SSA [4,5,6,7]. In most of these studies, the contamination by *A. flavus* was correlated with the levels of AFs in the feeds or feed ingredients. Aflatoxins are classified as group 1 carcinogens [8]. Aflatoxin B1 is the most prevalent of the AFs and in many animal species such as poultry, AFB1 has been reported to cause reduced growth, immunosuppression, and increased mortality, causing great economical losses [9].

*Fusarium verticillioides* and *F. proliferatum* are the main producers of FBs and have been reported to be prevalent in feed and feed ingredients such as maize from SSA [10,11,12,13]. Fumonisin B1 (FB1) was reported as the most prevalent FBs in feeds from SSA [14]. It is structurally similar to sphingosine, which is a constituent of various sphingolipids such as sphingomyelins, ceramides, cerebrosides, glycolipids, and ganglioside compounds found in the cell membranes of certain organs, which are particularly dense in nervous system tissues. This structural similarity with sphingosine has been suggested to cause FB1 to compete with sphingosine in sphingolipid metabolism, resulting in its toxicity [15]. Toxicity due to FBs was linked with leukoencephalomalacia in horses, pulmonary edema in pigs, hepatotoxicity in rats, and esophageal cancer in humans [16,17].

The growth of fungi and their ability to produce mycotoxins have been shown to be complex processes regulated by genetic mechanisms and affected by various environmental stimuli such as pH, temperature, relative humidity of the atmosphere, moisture, water activity, light, aeration, level of atmospheric gases as well as nutritional and biological factors. The production of AFs by *A. flavus* was shown to be the highest at temperatures of 28 °C or 30 °C, depending on the strain [18,19], whereas *F. verticillioides* produced FB1 at 15 °C in the soybean-based medium after 7 days of incubation [20]. The duration of inoculation for the maximum production of AFs by *A. flavus* was about 4 days in the coconut milk-derived liquid medium, between 4 and 8 days in yeast extract sucrose medium, and 21 days in peanut meal extract agar [18,21,22]. For the production of FBs, one study showed that peak production was reached after 5 weeks of inoculation of cracked maize with the *F. verticillioides* strain [16]. The substrate used has been shown to affect fungal growth and mycotoxin production, and *A. flavus* cultured on potato dextrose agar produced more AFs than in potato dextrose broth [19]. Higher levels of FBs were produced in coarsely cracked maize when compared to whole maize kernels or maize flour inoculated with the same *F. verticillioides* strain [16]. Light conditions such as yellow and green light enhanced the production of FBs by *F. verticillioides* strains when compared to the dark [17,23]. A moisture content of 50% resulted in the high production of FBs in coarsely cracked corn inoculated with *F. verticillioides* [16]. Variability of growth and mycotoxin biosynthesis was found to be inherent to the *A. flavus* strain and under similar conditions, the strains produced a different range and levels of mycotoxins [22,24].

The high cost and large quantities of mycotoxins required to evaluate the effects of animal exposure have been a hindrance to study the in vivo toxicities of most mycotoxins, particularly in low-income countries where the burden is the highest. There is a need to be able to maximize the laboratory production of mycotoxins for use in relevant studies including the in vivo efficacy testing of candidate mycotoxin detoxifiers to be used as feed additives. Several studies have also evaluated the toxicological properties in animals using commercially available purified AFs or FBs, which may not accurately represent the naturally produced mycotoxins that are consumed by farm animals under field conditions [25,26,27,28].

The aim of this work was therefore to optimize the production of AFs and FBs under laboratory conditions using local fungal strains and to produce sufficient quantities that can be used in experimental exposure studies of farm animals. The influences of strain, substrate, and incubation time on the production of AFs and FBs were tested. Additionally, the production of FBs by *F. verticillioides* was examined under red, yellow, and white light conditions.

## 2. Materials and Methods

### 2.1. Reagents and Chemicals

Potato dextrose agar (PDA) (HIMEDIA^®^), yeast extract sucrose agar (YESA) (HIMEDIA^®^), glucose yeast peptone agar (GYPA) (HIMEDIA^®^), sucrose, yeast extract, potassium dihydrogen phosphate (KH_2_PO_4_), calcium carbonate (CaCO_3_), magnesium sulfate (MgSO₄), and HPLC-grade methanol (MeOH) were obtained from Sigma Aldrich (St. Louis, MO, USA). Direct competitive enzyme-linked immunosorbent assay (ELISA) kits (AgraQuant^®^ Aflatoxin B1 and AgraQuant^®^ Fumonisin) obtained from Romer Labs (Singapore) were used to quantify the toxins produced. Corn-based reference materials for AFs or FBs were from Biopure^®^, Romer Labs, Inc. (Tulln, Austria).

### 2.2. Aspergillus flavus and Fusarium verticillioides Fungal Strains

Known high AF producing fungal strains of *A. flavus* (17s, 121365s, and 86s) and FBs producing fungal strains of *F. verticillioides* (K52, K826, and K81C) were kindly provided by the Mycology and Mycotoxin Laboratory, University of Nairobi, Kenya. The reference codes in brackets are the codes of the fungal strains as held in the laboratory. The AF-producing strains were previously isolated from maize collected from farmers in the eastern and Rift Valley regions of Kenya [2]. The isolates were morphologically, culturally, and molecularly identified before being preserved on silica gel at the laboratory.

### 2.3. Growth Media

The PDA was used for the initial sub culturing of the fungi and was prepared according to the manufacturers’ instructions.

Maize kernels, YESA, and yeast extract sucrose (YES) broth were used for the production of AFs, whereas cracked maize, GYPA, and V-8 juice broth were used for the production of FBs.

### 2.4. Production Methods for Aflatoxins and Fumonisins

The method described by Okoth et al. (2018) [29] for the production of AFs was used with some modifications.

The different fungal strains were first grown on 9-mm PDA plates amended with 2 mL/L lactic acid and the plates were incubated at 25 °C for seven days to obtain heavily sporulating cultures.

#### 2.4.1. Production Methods for Aflatoxins and Fumonisins in Agar Media

For the production of AFs, the agar media were prepared by weighing 40.5 g of YESA into media bottles, 1000 mL of distilled water was added to each bottle, the mixtures were thoroughly shaken, and then autoclaved for 30 min at 121 °C. Twenty-five mL of the media were dispensed into 9-cm-diameter Petri plates, cooled, and inoculated with three fungal plugs from the *A. flavus* strain previously grown on 9-mm PDA plates or one plug from each *A. flavus* strain in the case of combined inoculations with all the strains. The agar cultures were incubated (Heraeus products, Minneapolis, MN, USA) at 29 °C for 21 days. After every seven days, fungal plugs were uniformly picked from three Petri plates, transferred to amber bottles, and kept at −20 °C until analysis.

For the production of FBs, 50.0 g of GYPA was used instead and the media were inoculated with *F. verticillioides* strains, incubated at ambient temperatures (22–25 °C) in growth chambers fitted with white light.

Controls for the production of AFs and FBs in agar media were treated the same way, except that they were inoculated with sterile distilled water instead of the fungal strains.

#### 2.4.2. Production Methods for Aflatoxins and Fumonisins in Maize Kernels

Maize media were prepared as follows: 50 g of locally obtained white maize kernels were placed in 250 mL flasks and 20 mL of sterile distilled water was added to each flask. The flasks were then covered with cotton wool and aluminum foil. The maize kernels were left to imbibe the water at room temperature overnight, autoclaved for 30 min at 121 °C, cooled, and then inoculated with three fungal plugs of the *A. flavus* strain previously grown on 9-mm PDA plates for the production of AFs. In flasks where the strains were combined, one plug from each *A. flavus* strain was used. The flasks were kept in the incubator operated at 29 °C for 21 days, mechanically shaken daily from the third day to uniformly distribute the inoculum and to prevent the maize kernels from clumping together.

The same procedure described for the production of AFs in maize was used for the production of FBs. However, coarsely cracked maize kernels were used instead. The maize kernels were inoculated with *F. verticillioides* strains and the cultures incubated in growth chambers kept at ambient temperatures between 22 and 25 °C. The chambers were fitted with white, red, or yellow lights.

After every seven days, three flasks were taken from each chamber and from the incubator, oven-dried at 60 °C overnight, and milled to a fine powder using a blender (Waring Products DIV., Torrington, CT, USA). The milled samples were transferred to amber bottles and kept at −20 °C until analysis.

Controls for the production of AFs and FBs in maize media were treated the same way, except that they were inoculated with sterile distilled water instead of the fungal strains.

#### 2.4.3. Production Methods for Aflatoxins and Fumonisins in Broth Media

Broth media (YES broth) for the production of AFs were prepared by weighing 4.0 g of yeast extract, 20.0 g of sucrose, 1.0 g of KH_2_PO_4_, and 0.5 g of MgSO₄ into a media bottle. Distilled water (1000 mL) was added, the mixture thoroughly shaken, autoclaved for 30 min at 121 °C, cooled, and 100 mL of the media transferred into 250 mL flasks. The media were inoculated with three fungal plugs from each of the *A. flavus* strains previously grown on 9-mm PDA plates. In flasks where the strains were combined, one plug from each *A. flavus* strain was used. The cultures were incubated on a rotary shaker operated at 150 rpm and at ambient temperatures (22–25 °C) for the 21 days of the experimental period. The flasks were covered with black polythene to avoid exposure to light and to provide dark conditions similar to those of the maize and agar cultures kept in the incubator.

The same procedure was used for the production of FBs in broth media. However, the broth media were prepared using 1.0 g of yeast extract, 3.0 g of CaCO_3_, 1.0 g of glucose, and 200 mL of V8 juice. The media were inoculated with *F. verticillioides* strains and incubated on the rotary shaker and were not covered with black polythene.

After every seven days, three flasks were taken from each experiment, filtered using four layers of gauze, transferred to amber bottles, and kept at −20 °C until analysis [19].

Controls for the production of AFs and FBs in broth media were treated the same way, except that they were inoculated with sterile distilled water instead of the fungal strains. The levels of AFB1 and total FBs (FB1, FB2, FB3) in the different growth media were screened using ELISA methods.

### 2.5. Experimental Design

Experiment 1 involved the inoculation of maize, agar, and broth with three *A. flavus* strains (17s, 121365s, and 86s), alone or in combination for the production of AFs and the inoculation of maize, agar, and broth with three *F. verticilliodes* (K52, K826, and K81C), alone or in combination for the production of FBs (FB1, FB2, FB3). In experiment 2, the culture materials were sampled after every seven days for the determination of the production levels of AFB1 or FBs. Experiment 3 involved the production of FBs by inoculating maize with the three strains of *F. verticilliodes*, alone or in combination, and incubating the cultures in chambers with white, yellow, or red lights.

### 2.6. Variables Evaluated

Concentrations of AFB1 or total FBs were determined in the samples collected from the different media (agar, maize and broth) that were incubated with different fungal strains, alone or in combination. The samples were collected at different time points during the experimental period to evaluate the time dependent production of the AFB1 or total FBs. Additionally, the total FBs were determined in the maize culture samples collected from the chambers with the three different light conditions (red, yellow, or white).

### 2.7. Analysis of Different Culture Media for Aflatoxin B1 or Total Fumonisins Using ELISA Methods

The sample preparation, extraction, and assay procedures were performed according to the protocol given by the manufacture of the ELISA kits (AgraQuant^®^ Aflatoxin B1 and AgraQuant^®^ Fumonisin, Romer Labs, Singapore). In brief, AFB1 and the total FBs were extracted from three replicates of a 5 g subsample of milled maize culture flour, 5 mL of filtered broth cultures, or 5 g YESA or GYPA plugs. The extraction solution (25 mL of 70/30 (*v*/*v*) methanol/water) was added to each sample and the mixture was shaken thoroughly on a rotary shaker for 3 min. The mixture was allowed to settle before filtering through Whatman No. 1 filter paper (Whatman International Ltd., Maidstone, UK). The filtrates for the analysis of the total FBs were diluted with distilled water in a ratio of 1:20 before analysis, whereas for AFB1, the filtrates were analyzed without further dilutions. A microplate reader with a 450 nm filter was used for the analyses. The quantitation range for AFB1 was 2 to 50 μg/kg with a limit of detection (LOD) of 2 μg/kg. For the total FBs, the quantitation range was 250 to 5000 μg/kg and the LOD was 200 μg/kg. For sample readings above the highest standards, the filtered extracts were further diluted with extraction solution (70% methanol 30% distilled water) for AFB1, whereas for the total FBs, the filtered extracts were further diluted with the extraction solution and diluted with distilled water and reanalyzed, taking the dilution factor into consideration. Quality control for each batch was assessed by the analysis of samples together with the corn-based reference materials for AFs or FBs.

### 2.8. Data Analysis

All statistical analyses were performed in the R software package [30]. The AFB1 or total FBs concentrations were presented as mean ± standard deviation (SD). Comparisons of AFB1 and the total FBs concentrations in the different media and light conditions (for total FBs) were conducted using a non-parametric Kruskal–Wallis test since according to the Shapiro–Wilk normality and Levene’s tests, the data did not meet the normality and homoscedasticity assumptions for the parametric analysis of variance (ANOVA). In cases where significant differences were observed (*p* values < 0.05), post hoc Dunn tests were performed to observe where the differences were situated.

## 3. Results and Discussion

### 3.1. Analysis of Aflatoxin B1 in the Different Media

Aflatoxin B1 production was influenced by the strain, duration of incubation, and the growth media used (Table 1). The highest AFB1 level of 12,550 ± 3396 μg/kg was detected in maize kernels inoculated with all the three strains of *A. flavus* for 21 days. Numerically higher AFB1 levels were detected in maize, agar, and broth cultures with the three *A. flavus* strains combined when compared to cultures with single strains. This study demonstrated that inoculation with more than one strain of fungi led to enhanced AFB1 production. Asurmendi et al. (2015) [31] also observed an increased production of AFB1 and reduced fungal growth when strains of *A. flavus* were inoculated with a pathogenic bacterium, *Listeria monocytogenes*, in Brewer’s grains meal medium. Intraspecific competition, however resulted into reduced levels of AFB1 produced in inoculums with both toxigenic and non-toxigenic *A. flavus* strains [32]. The ability of non-toxigenic fungi to outcompete toxigenic fungi has been used as a biocontrol to prevent mycotoxin production [33]. Diverse mycoflora usually present on plants and in growth medium may lead to complex interactions among species such as competition for nutrients and space to grow. In such scenarios, the production of secondary metabolites, especially mycotoxins, can be used as a chemical antagonist to outcompete other species, although there is still a lack of enough evidence to support this view [34,35]. 

In the present study, higher levels of AFB1 were detected in agar cultures inoculated with strain 86s and sampled after 7, 14, and 21 days, compared to maize and broth inoculated with the same *A. flavus* strain. After 21 days, significantly higher AFB1 productions were observed in agar cultures when compared to broth cultures (*p* = 0.002) (Table 1). These results agree with studies by Wang et al. (2017) [36] who reported a significantly higher production of AFs in potato dextrose agar (23 μg/kg) compared to potato dextrose broth (5 μg/kg) inoculated with the same *A. flavus* strain. In another study, *A. flavus* isolates produced more AFB1 in Czapek yeast agar (501 ± 81 μg/kg) compared to the corn extract media (3 ± 1 μg/kg) after 21 days of incubation at temperatures of 30 °C and water activity of 0.96 [37]. *A. flavus* produced more AFB1 in processed rice or shelled peanut kernels compared to rice paddy, commercial media, and peanut media [38,39,40]. Production of AFs by *A. flavus* was shown to be influenced by the source of carbon in the growth media, and sucrose resulted in the highest toxin levels compared to other carbon sources such as starch [41]. In the latter study, the high production of AFs in media with sucrose was attributed to sucrose being a simple sugar, thus capable of supporting growth and the production of AFs. These studies imply that more than one substrate is required when evaluating the toxin-producing ability of a fungal strain in order to not miscalculate its toxin-producing ability.

For all of the strains inoculated in the three media (maize, agar, and broth) the AFB1 production increased with an increase in the incubation time, reaching peak production (12,550 ± 3397 μg/kg) after 21 days, with the exception of strain 17s inoculated in maize where peak production was after 14 days (Table 1). The first week was characterized by growth and colonization of the substrates by the different fungal strains and by the end of the second week, the substrates were fully colonized. In other studies, an AF yield of 436 μg/kg was detected after six days of inoculation of *A. flavus* isolates in modified YES medium and an AF yield of 866 μg/kg was seen after 30 days of inoculation in a rice paddy [18,42]. The mean AFB1 levels of 2115 ± 249 μg/kg were detected in potato dextrose agar cultures after 7 days of inoculation with the *A. flavus* strain isolated from maize grains [32]. Jamali et al. (2013) [43] reported AFB1 concentrations of up to 321,560 μg/kg in YES medium after seven days of inoculation with *A. flavus* strains isolated from the soils of pistachio orchards. Maximum AFs (53,710 ± 8790 μg/kg) were produced after three weeks of inoculation of peanut meal extract agar with the *A. flavus* strain isolated from raw peanuts [21]. The variations in the AF concentrations in the various studies were attributed to differences in the incubation period, origin of the strains used, and the differences in the eco-physiological conditions used in the different experiments.

### 3.2. Analysis of Total Fumonisins in Different Culture Materials Inoculated with F. verticillioides Strains and Incubated in Chambers with White Light or on a Rotary Shaker

Maize, agar, and broth media inoculated with the three strains of *F. verticillioides* singly or combined under white light conditions and ambient temperatures of 22 to 25 °C resulted in the production of FBs with levels depending on the duration of incubation as well as the media used (Table 2). The highest total FBs (FB1 + FB2 + FB3) levels of 117,496 ± 57,961 μg/kg substrate were detected in maize cultures inoculated with all the three strains of *F. verticillioides* for 21 days. Previous studies have shown that in addition to abiotic factors, biotic factors such as the presence of other microorganisms influenced the fungal growth and toxin production [31,32]. In the present study, after 21 days of inoculation with the strains of *F. verticillioides*, the mean FBs level was significantly higher in the cracked maize compared to broth (*p* = 0.003). Similarly, higher production of FBs in the maize patties compared to liquid cultures were reported [44,45,46]. The highest yields of FB1 (3,000,000 to 4,000,000 μg/kg of substrate) were obtained when highly toxigenic strains of *F. verticillioides* isolated from feeds were grown on coarsely cracked maize with 50% water content at 21 °C for five weeks [16]. In the latter study, lower levels of FB1 were detected in maize flour, whole maize, and rice, which was attributed to the ease of the fungus to access nutrients in cracked maize compared to maize left intact or rice. In addition, oxygen was more available in cracked maize compared to maize flour. These results suggest that a given strain of toxigenic fungi may produce varied levels of mycotoxins depending on the substrate.

There was an increase in the levels of total FBs in relation to the incubation time, reaching peak production after 21 days with the exception of strain K52 inoculated in broth media where peak production was after 14 days (Table 2). After 7 days, FBs were only detected in broth cultures with the K52 strain and maize cultures with the K826 strain, whereas the agar cultures had no detectable FBs. Similar to the production of AFB1 by *A. flavus*, the first 7 days were characterized by the growth and colonization of the various substrates by the different *F. verticillioides* strains and by the end of the 14 days, the substrates were fully colonized. Production of FB1 at 20 °C and 25 °C started after two weeks of inoculation and continued during the stationary phase, reaching a maximum yield (17,900,000 μg/kg) after 13 weeks before starting to decrease [47]. The decrease in FB1 levels after 13 weeks was attributed to the probable enzymatic cleavage of the main compound or conversion to other related compounds or both. In another study, lower levels of FB1 were detected in a liquid medium with the production of FB1 beginning after 3 days of inoculation and reaching a maximum (73 μg/mL) after 29 days [48].

### 3.3. Analysis of Total Fumonisins in Maize Culture Materials Inoculated with F. verticillioides Strains and Incubated in Chambers with White, Red, and Yellow Lights

**Figure 1:**
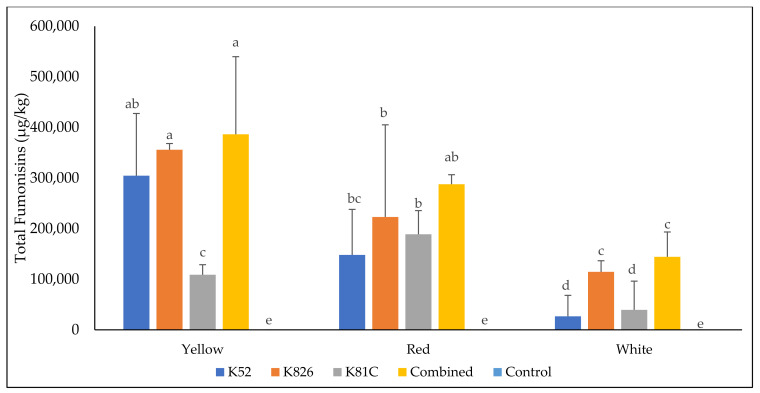
Total fumonisin (FB1, FB2, FB3) production in maize media after 21 days of inoculation with different *F. verticillioides* strains and incubated in chambers fitted with white, red, or yellow lights. Values are the means of three replicates ± standard deviation. Means with different letters are significantly different (*p* < 0.05) according to the post hoc Dunn test.

White, red, and yellow light conditions influenced the production of FBs by the different strains of *F. verticillioides* in maize cultures (Figure 1). After 21 days of inoculation, the mean FBs levels were significantly higher in maize incubated in growth chambers fitted with yellow light when compared to white light (*p* = 0.017). The highest FBs levels of 386,534 ± 153,303 μg/kg were observed in maize cultures inoculated with all three strains of *F. verticillioides* and kept under yellow light conditions for 21 days. Yellow light conditions also resulted in the highest FBs yield after 14 days (42,997 ± 1554 μg/kg) and 7 days (6115 ± 833 μg/kg) of inoculation of maize cultures with the K826 strain. Red light conditions enhanced the production of FBs when compared to the white light conditions, and after 21 days, maize inoculated with the K826 strain under red light conditions had significantly higher levels of FBs compared to maize inoculated with the same strain under white light conditions (223,144 ± 182,031 μg/kg versus 114,604 ± 21,951 μg/kg, *p* = 0.040). Matić et al. (2013) [17] demonstrated that all light conditions enhanced the production of FBs by *F. verticillioides* strains when compared to darkness. In the study, the enhanced production of FBs under different light conditions was attributed to light being able to stimulate the secondary metabolism of fungi, especially mycotoxin production. Wavelengths from red to blue were observed to increase FBs biosynthesis in *F. verticillioides* strains by up to 150% and in *F. proliferatum* strains by 40% compared to darkness [23,49]. Fanelli et al. (2016) [50], in their review, noted that *F. verticillioides* and *F. proliferatum* grew better and produced more FBs under light conditions.

Similar to the current study, white light conditions had the least influence on the production of FBs by *F. proliferatum* [49]. White light conditions increased FB1 and FB2 productions by only 3-fold whereas red by 40-fold, blue by 35-fold, royal blue by 20-fold, green by 10-fold, and yellow by 5-fold when compared to incubation in the dark. Therefore, light conditions are important factors to consider when evaluating the production of FBs by different *Fusarium* strains.

## 4. Conclusions

This study demonstrated that high yields of aflatoxins and fumonisins can be obtained in maize cultures and these large quantities can be used for animal trials to evaluate the effects of these mycotoxins on animal health and productivity, their transfer to animal source foods, and to study the in vivo efficacy of candidate mycotoxin detoxifiers used as feed additives. The study also demonstrated that the substrate, strain of fungi, incubation time, and light conditions as well as their interactions significantly influenced the production of mycotoxins. For each experiment aiming for the production of large quantities of mycotoxins, the optimal conditions for the strains used need to be identified. Further research should be conducted to evaluate the differential effects of environmental and biological conditions on the growth and production of mycotoxins by different fungal strains so as to not underreport the total mycotoxins produced.

## Figures and Tables

**Table 1:** Aflatoxin B1 production in the three media inoculated with various *A. flavus* strains.

Isolate	Media	Aflatoxin B1 (μg/kg) (Mean ± SD), n = 3		
		Day 7	Day 14	Day 21
17s	Maize	799 ± 241 ^b^	2945 ± 2066 ^bc^	177 ± 217 ^d^
121365s		837 ± 439 ^b^	4373 ± 348 ^ab^	1767 ± 1915 ^bc^
86s		725 ± 209 ^bc^	4304 ± 2513 ^ab^	10,255 ± 3763 ^a^
Combined		1477 ± 1630 ^a^	5630 ± 1256 ^ab^	12,550 ± 3397 ^a^
Control		ND	ND	ND
17s	Broth	259 ± 449 ^c^	642 ± 904 ^c^	510 ± 884 ^c^
121365s		545 ± 239 ^bc^	1786 ± 983 ^c^	2606 ± 264 ^b^
86s		560 ± 63 ^bc^	1399 ± 125 ^c^	2441 ± 877 ^b^
Combined		854 ± 30 ^b^	2392 ± 1235 ^bc^	2713 ± 3142 ^b^
Control		ND	ND	ND
17s	Agar	560 ± 320 ^bc^	2475 ± 1582 ^bc^	6571 ± 5693 ^ab^
121365s		649 ± 222 ^bc^	4090 ± 240 ^ab^	6570 ± 5690 ^ab^
86s		776 ± 83 ^bc^	5588 ± 2673 ^ab^	11,753 ± 9250 ^a^
Combined		1913 ± 756 ^a^	8551 ± 6393 ^a^	12,158 ± 6809 ^a^
Control		ND	ND	ND

ND; Not detected (<2 μg/kg), Values are the means of three replicates ± standard deviation (SD). Values within the same column not sharing a common superscript differed significantly (*p* < 0.05) according to a post hoc Dunn test.

**Table 2:** The total fumonisin levels in three media inoculated with various *F. verticillioides* strains and incubated in chambers fitted with white light or on a rotary shaker.

Isolate	Media	Total Fumonisins (μg/kg) (Mean ± SD) n = 3		
		Day 7	Day 14	Day 21
K52	Maize	ND	10,253 ± 16,188 ^ab^	48,735 ± 6473 ^ab^
K826		1855 ± 3213 ^a^	19,932 ± 15,940 ^a^	71,374 ± 31,338 ^ab^
K81C		ND	15,080 ± 23,671 ^ab^	65,872 ± 26,719 ^ab^
Combined		ND	26,321 ± 14,004 ^a^	117,496 ± 57,961 ^a^
Control		ND	ND	ND
K52	Broth	729 ± 1262 ^a^	4072 ± 7052 ^b^	2786 ± 4010 ^d^
K826		ND	1855 ± 3213 ^bc^	6533.50 ± 5121 ^c^
K81C		ND	4148 ± 3858 ^b^	6590.33 ± 5911 ^c^
Combined		ND	5140 ± 1188 ^b^	21,485 ± 3118 ^bc^
Control		ND	ND	ND
K52	Agar	ND	921 ± 1596 ^c^	39,395 ± 6463 ^b^
K826		ND	787 ± 1363 ^c^	32,332 ± 15,355 ^bc^
K81C		ND	934 ± 1430 ^c^	49,359 ± 84,423 ^ab^
Combined		ND	790 ± 1164 ^c^	6686 ± 1057 ^c^
Control		ND	ND	ND

ND; Not detected (<200 μg/kg). Values are the means of three replicates ± standard deviation (SD). Means within a column with different superscript are significantly different (*p* < 0.05) according to a post hoc Dunn test.

## Data Availability

The data presented are available upon reasonable request.
